# Development of a synthetic library of humanized nanobodies for targeted IL-6 inhibition

**DOI:** 10.3389/fbioe.2024.1440150

**Published:** 2024-07-23

**Authors:** Lei Wang, Jiayi Dong, Chenlu Wu, Chenyue Yan, Chong Bi, Chengnan Xu, Yiling Wu, Wenyun Zheng, Xingyuan Ma

**Affiliations:** ^1^ State Key Laboratory of Bioreactor Engineering, School of Biotechnology, East China University of Science and Technology, Shanghai, China; ^2^ Shanghai Key Laboratory of New Drug Design, School of Pharmacy, East China University of Science and Technology, Shanghai, China

**Keywords:** nanobody, humanization, stability, synthetic library, phage display, interleukin-6, blocking antibody

## Abstract

Interleukin-6 (IL-6) is a cytokine that can bind to IL-6 receptor and induce pleiotropic effects. It serves as a critical biomarker, involved in inflammation amplification, tumor progression, and many other disease developments. Nanobodies, featuring small structure and high affinity, are a powerful and versatile tool in medical diagnostics and therapeutics. Here, based on a scaffold optimized for humanization and stability, we developed a synthetic phage display library that rapidly generated high-affinity and humanized nanobodies, negating the need for animal immunization. Using enhanced green fluorescent protein (eGFP) as a benchmark, we demonstrated that the library produced humanized nanobodies with high function and great intracellular stability. The library was then subjected to screening against IL-6. We identified a standout nanobody, NbL3, which exhibited high affinity (22.16 nM) and stability and significantly inhibited IL-6-enhanced migration on the human breast cancer cell MCF-7 at a relatively low concentration. NbL3’s strong blocking activity provides a promising therapeutic alternative for the IL-6-targeted intervention strategy, underscoring the broader potential of our synthetic library as a versatile platform for the development of humanized nanobodies against multiple antigens.

## 1 Introduction

Interleukin 6 (IL-6), secreted by a variety of cell types, is a cytokine with pleiotropic effects that are involved in the detection of infections, inflammation, and other diseases. IL-6 has been found to be significantly elevated in severe diseases such as sepsis, acute respiratory distress syndrome (ARDS), and even COVID-19 (McElvaney et al., 2021), most recently. In addition to its capability of amplifying inflammation, it drives cancer progression by activating multiple signaling pathways and augmenting the expression of anti-apoptotic proteins (Briukhovetska et al., 2021). Translational research of IL-6 biology into therapies targeting its pivotal pathway has been progressing for more than a decade, and IL-6 inhibitors have been shown to be effective in treatments of chronic arthritis (JIA), Takayasu arteritis and giant cell arteritis (GCA), cytokine release syndrome (CRS), and other severe diseases (Choy et al., 2020). The administration of monoclonal antibodies, tocilizumab and siltuximab (Tanaka et al., 2018) or the development of nanobodies, vobarilizumab (Labrijn et al., 2019) have shown important significance in clinical applications.

Heavy chain antibodies (HCAbs) were first discovered by professor Raymond Hamers-Casterman in dromedary camels (Hamers-Casterman et al., 1993). They are distinguished by the absence of CH1 domains and the requirement for light chain pairing. Hence, they only consist of two heavy chains, each containing a variable antigen-binding domain (VHH), referred to as single domain antibody (sdAb) or nanobody (Nb). Nanobodies have an extended complementarity-determining region 3 (CDR3) loop that interacts with framework 2 (FRW2) to maintain structural stability (Muyldermans, 2013). Within the FRW2, there are four hydrophilic hallmarks (F37, E44, R45, and G47) (the international ImMunoGeneTics information system, IMGT (Dondelinger et al., 2018) that are pivotal for soluble and monomeric expression. Nanobodies, with a molecular weight of approximately 15 kDa, are characterized by high solubility and stability, strong permeability, low immunogenicity, and easy tailoring (Yu et al., 2023). These properties have led to their extensive applications, including fundamental biological research, diagnostics, and therapeutics (Jin et al., 2023). For example, they are applied for improving crystallographic stability and resolution as chaperone proteins (Condeminas and Macias, 2024), for facilitating the detection of microorganisms (Salvador et al., 2019), or for tumor treatment as targeting drugs (Kunz et al., 2023). Although they possess many desirable properties, the non-humanized nanobodies may increase the risk of inducing undesirable immune responses when utilized in clinical use, such as anti-drug antibody (ADA) (Rossotti et al., 2022). Therefore, humanization represents a critical step in alleviating immunogenicity and advancing the application of nanobodies.

Phage display has been widely applied in antibody screening due to its easy amplification and affordability (Bozovičar and Bratkovič, 2019), leading to the discovery of numerous therapeutic antibodies for clinical development (Frenzel et al., 2016). By creating a synthetic nanobody library and displaying these nanobodies on the phage coat protein pШ or pⅧ (Jaroszewicz et al., 2022), antigen-specific nanobodies can be rapidly identified with the assistance of *in vitro* screening. Compared to immune and naïve libraries, the synthetic library is emerging as a charming alternative to circumvent animal immunization for the generation of nanobodies, which are accessible for most laboratories (Liu and Yang, 2022). Additionally, the construction of humanized nanobody library makes it possible to eliminate humanized nanobodies that are tricky to express in bacteria during *in vitro* screening, thereby accelerating the process of nanobody humanization.

**FIGURE 1:**
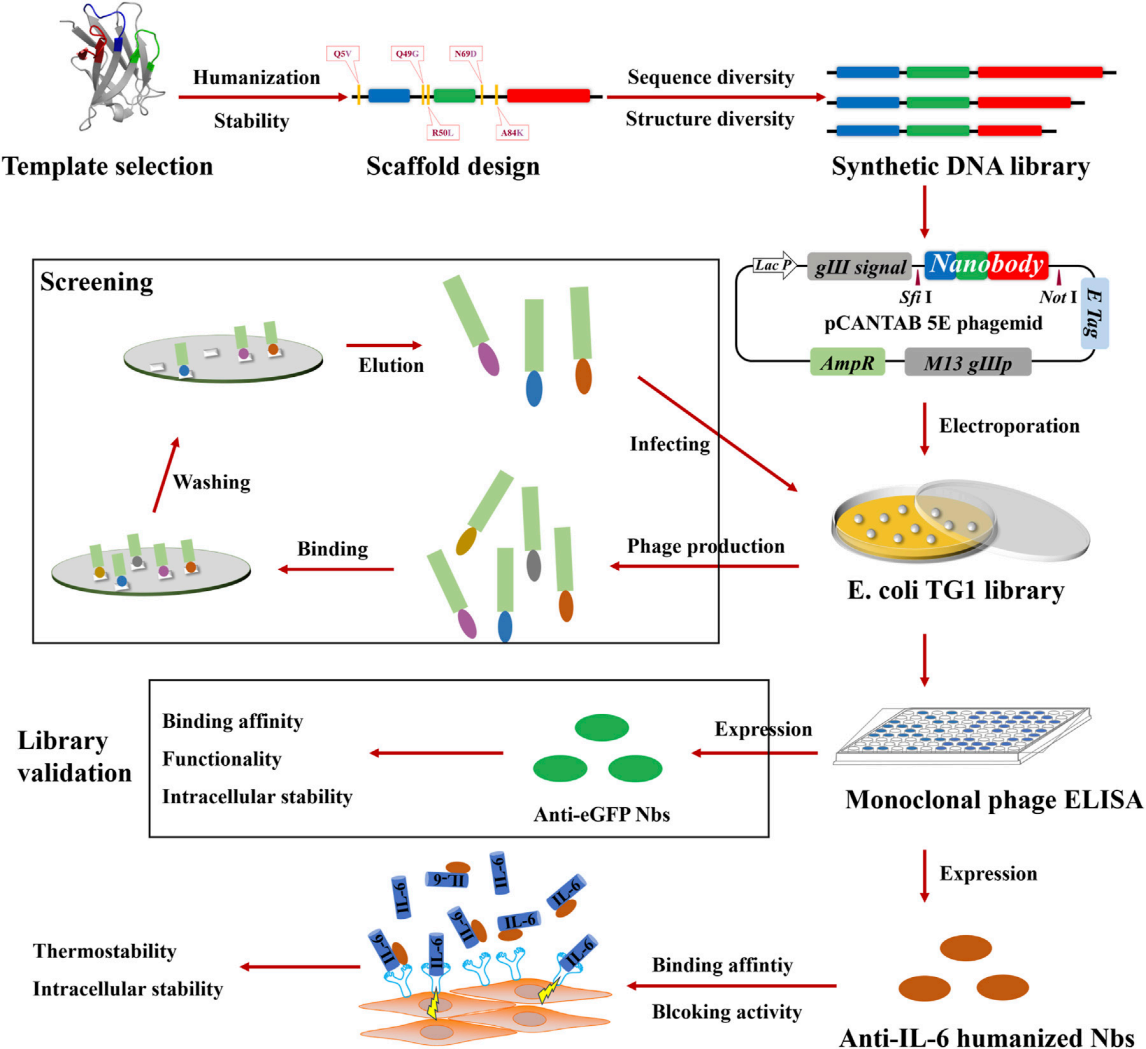
Schematic illustration of the construction of the synthetic library and the generation of IL-6 blocking antibodies. As a first step, a synthetic library of humanized nanobodies was constructed, which involved template selection, scaffold design, DNA library synthesis, and the electroporation of *E. coli* TG1. Next, library validation was carried out, which involved screening against eGFP, monoclonal phage ELISA for positivity verification, and characterizations of anti-eGFP nanobodies. Finally, anti-IL-6 humanized nanobodies were selected, following the assessment of blocking activity and the characterization of stability.

In this work, we developed a synthetic library to generate humanized nanobodies targeting IL-6 (Figure 1). First, a well-resolved nanobody with exceptionally high affinity was preferred. To generate a nanobody scaffold, it was designed to reduce the distance between the sequences of llama and human VH3 while simultaneously to improve stability. We then confirmed the thermostability and intracellular stability of this humanized nanobody scaffold as robust to construct a library. To increase structure diversity, the relationship between CDR3 and FRW2 was analyzed, and CDR3 length variation was decided. To introduce amino acid diversity into CDR loops without the unpredictable risk of scaffold destabilization, loop structure conserved sites are defined to maintain the hydrophobic core, and non-conserved sites are randomized according to the sequence alignment of naturally occurring nanobodies. With the information, a synthetic library of humanized nanobodies was constructed, and an effective capacity of 3.7 × 10^9^ was obtained. The synthetic library was screened against enhanced green fluorescent protein (eGFP). Anti-eGFP nanobodies that enhanced the fluorescence intensity of eGFP with great intracellular stability were selected, which demonstrates that the library produced humanized nanobodies with high function. Subsequently, nanobodies against IL-6 were selected and evaluated. Binding affinity measurements indicated that they could reach the 10–100 nM range of binding strength. Stability characterizations showed that they possessed good thermostability *in vitro* and high intracellular stability, as expected from the previous design. Further examination of the blocking activity showed that they exhibited a significant inhibition of IL-6-enhanced migration on the human breast cancer cell MCF-7. This thus indicates a promising therapeutic alternative for the IL-6-targeted intervention strategy. Our library permits the development of humanized nanobodies and underscores a broader potential to screen against multiple antigens.

## 2 Materials and methods

### 2.1 Materials and instruments

*Escherichia coli* TG1 electrocompetent cells and were provided by LGC Biosearch Technologies (England). M13KO7 helper phages were purchased from NEB (United States). The restriction endonucleases of QuickCut™ *Sfi* Ⅰ, *Not* Ⅰ, and T4 DNA ligase were supplied by Takara (Japan). One Step Cloning kit was commercially available in Yeasen (China). Human embryonic kidney (HEK) 293T and Human breast cancer (MCF-7) cells were obtained from the Type Culture Collection Committee of Chinese Academy of Sciences (China). Transfection reagents were prepared with PEI linear polyethylenimine (Sigma, Germany). 96-well polystyrene high bind microplates were acquired from Corning (China). Protein purification was carried out with ClearFirst-1000L (Flash, China). *E. coli* bacteria crushing was performed using High pressure cell crusher (Union-Biotech, China). Thermostability was monitored by circular dichroism spectroscopy (Applied photophysics, United Kingdom). Live fluorescent images were taken using an Olympus XDS-200C microscope (Japan) and a laser scanning confocal microscope (Nikon A1R+, Japan). Intracellular TagBFP signal was evaluated using FACSCalibur Flow Cytometer (BD, United States). Absorbance determination was performed on Synergy H1 microplate reader (BioTek, United States).

### 2.2 *In silico* analysis

All nanobody Sequences in this study were numbered by the IMGT scheme. Sequence alignment was performed using MEGA 11. Before alignment, sequences were pre-processed, which included, but was not limited to removing the redundant sequences by CD-HIT and eliminating the sequences that were not from the camelid. Structure superposition was carried out using Pymol. The dataset consists of 40 nanobody-antigen complexes obtained in 2022 from Protein Data Bank (PDB) (Supplementary Material). They were selected based on the several criteria. First, the CDR3 lengths of nanobodies were 8, 11, or 14 aa. Second, the crystal structures of the complexes were intact and determined with a great resolution around 3 Å. Next, the sequences were reduced based on sequence identity (similarity > 90%). Finally, they were not allowed to bind the same antigens.

### 2.3 Thermostability by circular dichroism (CD) spectroscopy

Nanobodies were diluted to 0.1–0.3 mg/mL in PBS (pH 7.4). A 1 mm pathlength rectangular quartz cuvette was used, and CD spectra were collected between the wavelengths of 180–260 nm at a ramp rate of 5.0°C/min over a temperature range of 25°C–95°C to obtain the optimal wavelength for monitoring secondary structure alteration. At the optimal wavelength of 215 nm, CD data were collected again over the same temperature range but at a ramp rate of 2.5°C/min. Raw ellipticity data (mdeg) was smoothed using Chirascan and converted to molar ellipticity (θ). Molar ellipticity was then used to calculate the fraction of folded protein. The reversibility was assayed by cooling the temperature down to 25°C.

### 2.4 Cell culture and transfection

HEK293T cells were cultured and maintained in DMEM complete medium. All nanobody-TagBFP (Nb-TagBFP) constructs were transfected into HEK293T cells. One day prior to transfection, 24-well plates were used and cells were plated at approximately 50,000 cells per well. Cells were incubated at 37°C, and 5% CO_2_ until the confluent reached 70%–90%. For transfection in each well, 1.0 μg plasmid Nb-TagBFP DNA (500 ng Nb-TagBFP plasmid and 500 ng DsRed plasmid) was diluted in 25 μL serum-free media. For functional identification of anti-eGFP nanobodies, an additional 500 ng EGFP plasmid was added. For the control group, Nb-TagBFP plasmid was substituted with TagBFP plasmid. 3 μL of 1 mg/mL PEI was dissolved in 25 μL serum-free media and mixed gently and incubated for 5 min at room temperature. The plasmid DNA and the PEI dilutions were mixed gently and incubated for 15 min at room temperature before being added to cells. Cells were incubated at 37°C, and 5% CO_2_ for 48 h prior to live fluorescence imaging.

### 2.5 Live fluorescence imaging in HEK293T cells

The cells transfected with fluorescent proteins were assessed using an Olympus XDS-200C microscope and a × 40 objective lens. DsRed signal served as a transfection control for the assessment of cellular morphology and the determination of fluorescence intensity. Nanobodies were binned as stable and unstable groups based on the observed character of the TagBFP signal. The grouping criteria were decided to capture the major differences between the groups, not detailed differences instead. Stable nanobodies were filled with TagBFP signal and mirrored DsRed signal. It should be noted that nanobodies with TagBFP signal that exhibited only minor and infrequent fluorescent puncta were also binned as the stable group. Nanobodies with absent TagBFP signal or major fluorescent puncta were binned as the unstable group. TagBFP signal in this study was pseudo-colored in green for easier observation.

### 2.6 Synthetic library construction

The designed structure diversity and sequence diversity were synthesized in the degenerate primers F-2, R-2, and R-3. As a first step, CDR1 and CDR2 were assembled with F-2 and R-2, while CDR3 was amplified with F-3 and R-3 by performing PCR. Subsequently, CDR1, CDR2, and CDR3 were assembled with F-1 and R-1 primers by performing overlap extension PCR (Supplementary Table S1), which generated a full-length DNA library with *Sfi* Ⅰ and *Not* Ⅰ enzymatic digestion sites. Each PCR procedure was performed within a few cycles, such as 20 or 25 cycles, to ensure fidelity. PCR protocol was available under the instruction of the High-Fidelity DNA Polymerase (Yeasen, China). The resulting DNA library was purified on columns of a DNA gel purification kit (Magen, China). The DNA library was digested and purified using a PCR purification kit, while the pCANTAB-5E phagemid was digested and purified using the DNA gel purification kit. The purified DNA library was ligated into the purified phagemid at 16°C overnight with T4 DNA ligase. The resulting ligation was purified again using the PCR purification kit. The purified ligation was transformed into electrocompetent *E. coli* TG1 cells with electroporation more than 50 times. The bacteria were immediately resuspended with 2xYT containing 2% glucose, and incubated with a horizontal shaker for 1 h at 37°C. Library size was calculated by serial dilution, and colonies on the plate were used to determinate the insertion rate and correctness rate. The recovered cells were plated on 140 mm 2xYT agar dishes (100 μg/mL ampicillin) and grown overnight at 37°C. The colonies were scraped and collected with 2xYT. The cells were centrifuged and resuspended with 2xYT, and stored in the presence of a 30% final concentration of glycerol with 1 mL aliquots at −80°C.

### 2.7 Preparation of eGFP and IL-6 antigen proteins

His tagged proteins were synthesized into pET24a vectors. The vectors were chemically transformed into *E. coli* BL21 (DE3). The bacterial cells, transformed with eGFP expressing vectors, were cultured in LB media at 37°C with shaking until OD_600_ reached 0.4–0.6. The induced expression of eGFP was achieved with a final concentration of 1 mM IPTG and accumulated at 28°C with shaking at 225 r.p.m. overnight. IL-6 expression was induced with a final concentration of 0.75 mM IPTG at 20°C for 24 h. The cells were harvested and resuspended in PBS (pH 7.9). The protein supernatant was obtained by crushing the cells under a pressure of 750 MPa. The cell debris was pelleted by centrifugation and the supernatant was purified over Ni-NTA columns. The protein was eluted with 100 mM imidazole and then dialyzed with PBS to remove imidazole. The purity was verified by SDS-PAGE. The purified protein was concentrated using 3,000 NMWL Amicon Ultra-15 (Millipore). The concentration was determined using a BCA protein assay kit.

### 2.8 Screening against eGFP and IL-6

1 mL aliquot of the library was diluted with 500 mL 2xYT-ampicillin and grown to OD_600_ of 0.5. The culture was superinfected with 2 × 10^12^ M13KO7 and incubated at 37°C for 60 min. Cell pellets were harvested by centrifugation and resuspended in 500 mL 2xYT-ampicillin-kannamycin and cultured at 37°C and 250 r.p.m. overnight. Phage supernatant was collected by centrifugation, and precipitated by adding 20% PEG6000 (v/v) solution, and incubated on ice for 1 h. Phage pellets were collected by centrifugation and resuspended in PBS (pH 7.4). Phage titers were estimated by infecting *E. coli* TG1 cells with serially diluted phages. A polystyrene high bind microplate was coated with antigen protein at 4°C overnight. The plate was washed with 0.1% PBST and blocked with 3% BSA for 2 h. After rinsing, > 10^12^ phage particles (for the first round) were added and incubated for 1 h. Wells were washed, and 0.2 M Glycine-HCl (pH 2.2) was added and incubated for 15 min. Eluted phages were neutralized with 1 M Tris-HCl (pH 9.1) and collected to amplify for the next round. For the subsequent rounds of panning, 10^11^ phage particles were added per well.

Monoclonal phage ELISA, from the dilution series of the last panning round, was performed to verify the positive monoclonal phages. Briefly, 96 colonies were picked into a 96-well plate and grown overnight. The culture was diluted into another 96-well plate and grown to OD_600_ of 0.5. M13KO7 phages were added and incubated without shaking to infect cells. The plate was spined and the supernatant was discarded. Pellets were resuspended in fresh medium and grown overnight. The plate was spined and the supernatant was transferred to a new 96-well plate. The phage supernatant was diluted with 3% milk and tested by ELISA. A 96-well polystyrene high bind microplate was coated with antigen protein and blocked with 3% milk, while the control plate was coated with BSA. After rinsing, the phage supernatant was added to each well in both phage ELISA plates and incubated for 1 h. Wells were washed and HRP-anti-M13 antibody (Sino Biological, China) was added at 1:40,000 in PBS Tween 0.1% + 3% milk and incubated for 1 h. Wells were washed and reaction was developed with TMB substrate solution (Solarbio, China) at room temperature for 15 min. The light absorption value of each well was measured at 450 nm after the termination using 1 M hydrochloric acid. The phage clones were verified positive if the absorption ratio of the experimental group to the control group was above 2.

### 2.9 Expression and purification of nanobodies

His- and HA-tagged nanobodies were PCR amplified and cloned into pET26b vectors using the One Step Clone kit. The vectors were transformed into *E. coli* BL21 (DE3) chemically competent cells. A single colony was inoculated into 10 mL of LB media containing kanamycin (50 μg/mL) and cultured on a horizontal shaker overnight at 37°C and 250 r.p.m. Next day, this preculture was amplified in 500 mL LB and cultured at 37°C with shaking until OD_600_ reached 0.4–0.6. The expression of nanobodies was induced by IPTG with a final concentration of 1 mM and protein accumulation was achieved at 28°C with shaking overnight. The nanobody supernatant was purified over Ni-NTA columns and eluted with 200 mM imidazole. The affinity-purified nanobodies were dialyzed with PBS to remove imidazole.

### 2.10 Binding affinity determination

A 96-well polystyrene high bind microplate was coated with antigen protein and blocked with 3% milk, while the control plate was coated with BSA. After rinsing, the nanobody with a serial dilution was added to each well and incubated for 1 h. Wells were washed and HRP-anti-HA antibody (Sino Biological, China) was added at 1:15,000 in PBS Tween 0.1% + 3% milk and incubated for 1 h. Wells were washed and reaction was developed as described in monoclonal phage ELISA.

### 2.11 Function identification of anti-eGFP nanobodies

For function identification *in vitro*, purified recombinant eGFP and the corresponding nanobody were adjusted to 12 μM by dilution in PBS (pH 7.4). Samples were mixed at equal proportions and incubated at room temperature for 1 h (protected from light). eGFP, mixed with an irrelevant nanobody, was used as a control. The mixtures were added to a 96-well microplate and the value at 490 nm was detected by the microplate reader. The relative fluorescence intensity was calculated as the absorption ratio of the experimental group to the control group. For function identification intracellularly, Nb-TagBFP, DsRed, and EGFP plasmids were co-transfected into HEK293T cells, while the substitution of Nb-TagBFP to TagBFP was taken as a control. The transfection protocol was described in the method of cell culture and transfection. After 48 h of transfection, the cells were harvested to run flow cytometry. The absolute fluorescence intensities of eGFP and DsRed were collected. The normalized fluorescence intensity of eGFP was acquired by normalizing the absolute fluorescence intensity of eGFP to DsRed.

### 2.12 Thermostability by indirect ELISA

Nanobodies were treated at 60°C or 37°C in a water bath for a series of hours (0, 0.2, 0.5, 1, 2, 3, 4, and 5 h at 60°C, and 0, 0.5, 1, 3, 6, 12, 24, and 48 h at 37°C). After thermal treatment, the nanobodies were incubated at 4°C overnight. A 96-well polystyrene high bind microplate was coated with antigen protein and blocked with 3% milk, while the control plate was coated with BSA. After rinsing, the nanobodies were added to each well and incubated for 1 h. Wells were washed and HRP-anti-HA antibody (Sino Biological, China) was added at 1:15,000 in PBS Tween 0.1% + 3% milk and incubated for 1 h. Wells were washed and reaction was developed as described in monoclonal phage ELISA.

### 2.13 Bacterial expression and confocal imaging

It is reported that fluorescent proteins might fold incorrectly in the periplasm of *E. coli* (Feilmeier et al., 2000). Therefore, we cloned nanobody sequences into the bacterial expression vector pET24a and was fused to mCherry fluorescent protein. Plasmid was chemically transformed into *E. coli* BL21 (DE3), and individual colonies were inoculated into 5 mL LB and cultured overnight at 37°C with shaking. 1 mL of each culture was diluted into 4 mL M9 minimal media, and IPTG was added to a final concentration of 0.25 mM to induce protein expression in the cytosol for 4 h at 37°C with shaking. 40 μL of induced bacteria was added to 3% M9-agar on a glass slide and covered with a coverslip. The bacteria were imaged with a Nikon A1R + laser scanning confocal microscope using a ×100 oil immersion objective lens. All images were taken under a consistent excitation condition.

### 2.14 Migration assay of MCF-7

Cell migration analysis was performed by wound healing assay. 4 × 10^5^ MCF-7 cells were seeded into a 12-well plate and grown in DMEM with 10% FBS to cover the well. The cell monolayer was scratched with a pipette tip and washed with PBS (pH 7.4). The scratched cells were cultured in DMEM with 1% FBS containing different concentrations of IL-6 only or IL-6 in combination with nanobodies for 24 or 48 h. The control group was cultured in DMEM with 1% FBS. All images were taken using an Olympus XDS-200C microscope on 0, 24, or 48 h. Wound healing rate (%) = [(wound area at 0 h) − (wound area at each time)] ∕ (wound area at 0 h) × 100%.

### 2.15 Statistical analysis

Statistical analysis was performed using Prism statistical analysis software. All data are expressed as mean ± SEM. Data were analyzed using a grouped two-way ANOVA analysis, followed by multiple comparisons. *p* values are indicated by asterisks as follows: *p < 0.05, **p < 0.01, ***p < 0.001.

## 3 Results

### 3.1 Rational design of the scaffold and randomization strategy

Poor expression ability in *E. coli* will severely affect display efficiency on phages, resulting in the loss of high-affinity candidates. Nanobodies derived from llama usually contain a single disulfide bond and can be efficiently expressed in the cytosol of *E. coli* with correct folding (Olichon and Surrey, 2007). Well-resolved crystal structures are indispensable to presenting the true CDR loop structures of nanobodies, which favors structural analysis. Therefore, we analyzed a large collection of nanobody-antigen complexes with measured binding free energy (Zavrtanik et al., 2018). The Nb6B9, an exceptionally high-affinity G-protein-coupled receptor (GPCR)-stabilizing nanobody originated from llama, enables β_2_-adrenoceptor (β_2_AR) crystallization with three distinct agonists (Ring et al., 2013). As reported by its creators, the expression of Nb6B9 varied and could reach a high level of 10 mg/L. A nanobody that inserts a medium length CDR3 (12 amino acid, aa) was described, and its binding surface was defined as the loop surface (Zimmermann et al., 2018). Similarly, the Nb6B9 arranges its CDR3 (14 aa) into the receptor cavity as an extended loop surface, leading to flexibility between the concave and convex surfaces. Thus, the nanobody Nb6B9 was considered a potentially robust template.

The four vital hallmark positions in FRW2, 42, 49, 50, and 52 (IMGT) are the major contributions to the high solubility of nanobodies compared to human VHs. There have been comprehensive reports describing the humanization of these positions. For example, full humanization of NbHuL6 and NbBcⅡ10 at hallmarks adversely affected their expression level, solubility, and binding affinity, while partial humanization of 49 and 50 resulted in a significant increase in stability (Vincke et al., 2009), which was consistent with other studies (Rossotti et al., 2022). As in the first step, Q49G and R50L substitutions were introduced into the template towards the nearest human germline gene, *IGHV3-53*.

**FIGURE 2:**
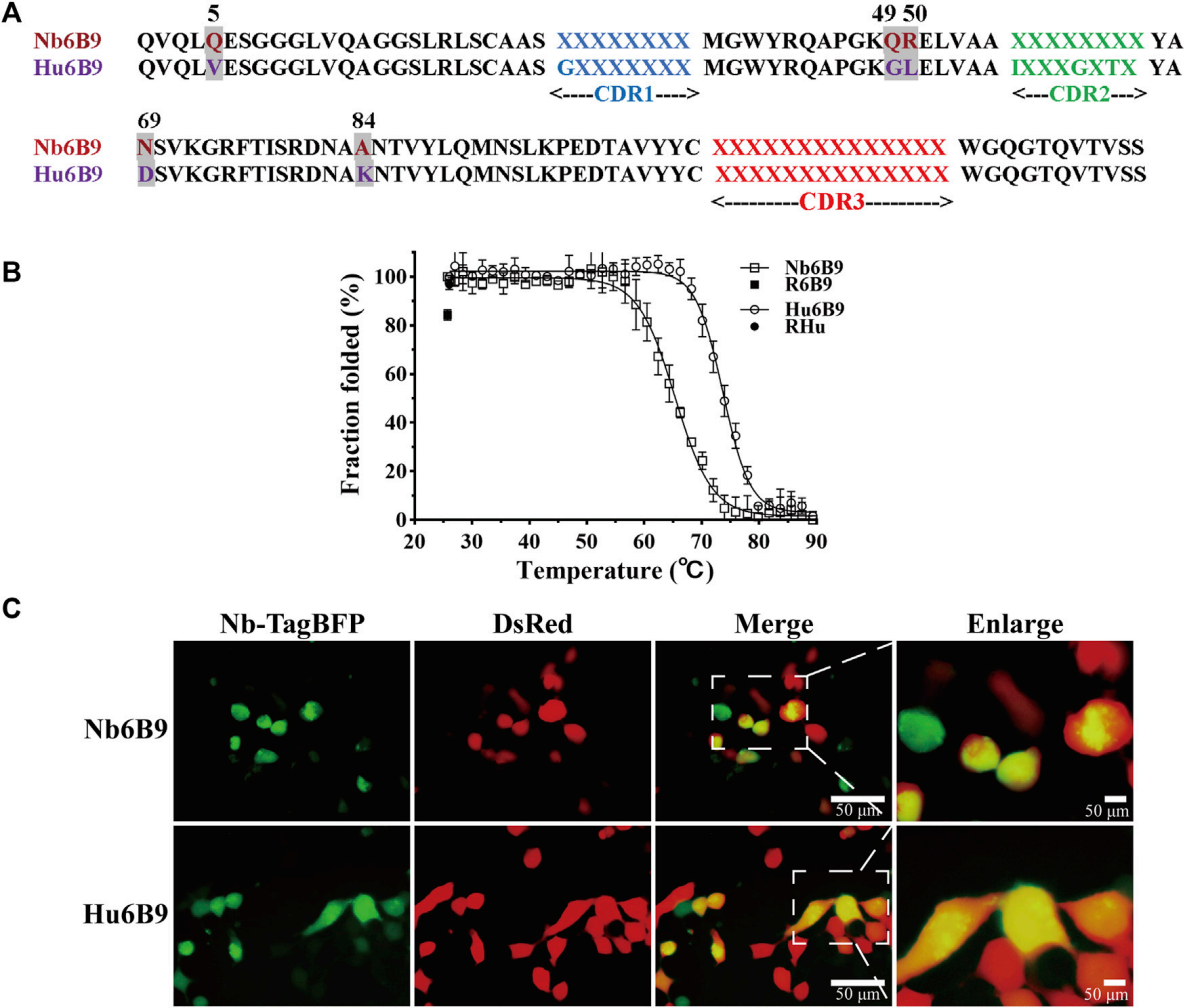
Overview of scaffold design and stability characterization. **(A)** Five substitutions (highlighted in gray) were introduced into the template Nb6B9, generating the scaffold Hu6B9. Blue, green, and red represent CDR1, CDR2, and CDR3, respectively. It is noteworthy that position 66 (IMGT) was included in CDR2 in this study. **(B)** Thermostability and thermal reversibility were monitored by circular dichroism spectroscopy. R6B9 and Rhu indicate the folded fraction of Nb6B9 and Hu6B9 after recovery to room temperature, respectively. **(C)** Representative images of Nb-TagBFP expression in HEK293T cells. Nb-TagBFP plasmid was transiently co-transfected with DsRed plasmid into HEK293T. Green signal is from Nb-TagBFP plasmid, while red signal is from DsRed plasmid.

In general, there are some consensus frameworks that bestow antibodies with great stability *in vitro* and intracellularly, so they represent robust scaffolds to accept numerous CDR loops (Knappik et al., 2000; Hoet et al., 2005; Moutel et al., 2016). Using comparative analysis, positional conservations were defined, and the partial consensus framework was generated. Framework mutagenesis identified that the partial consensus framework was successfully applied to rescue intracellular stability in a large group of unstable nanobodies (Dingus et al., 2022). To further humanize the template and achieve stable expression, the positional residues of the highest conservation were distilled from the partial consensus framework, in parallel with the consideration of improving similarity to human VH3. Hence, three more residues were altered, eventually resulting in five substitutions in the scaffold (designated as Hu6B9) (Figure 2A).

To test whether the Hu6B9 scaffold was robust to construct a library, stability *in vitro* and intracellularly was characterized by circular dichroism (CD) and live imaging in human embryonic kidney 293T (HEK293T) cells. The Hu6B9 scaffold exhibited a high melting temperature of 73.7°C and an excellent thermal reversibility (Dumoulin et al., 2002) of 96.7%, which corresponds to an increase of 8.4°C and 12.4% compared to the template (Figure 2B). Additionally, the scaffold exhibited a mild improvement in intracellular stability in HEK293T cells (Figure 2C).

**FIGURE 3:**
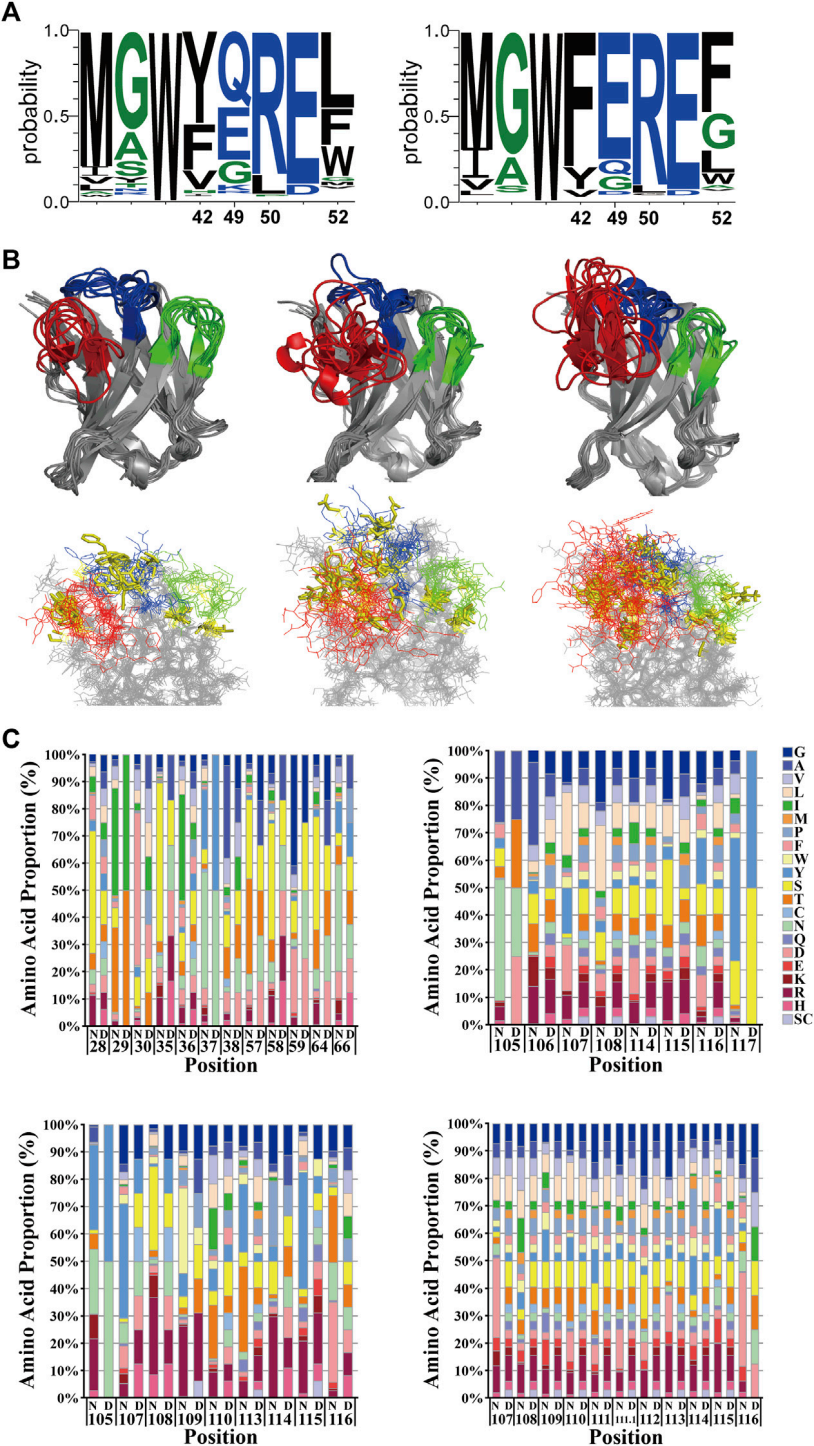
Rational design of the randomization strategy. **(A)** The relationship between hallmarks in FRW2 and CDR3 lengths. Y42 and L50 occurred in CDR3 lengths ≤ 14 aa (left), while F42 and F52 appeared in CDR3 lengths > 14 aa (right). Created by WebLogo 3.7.12. **(B)** structural superposition with three-dimensional views. Nanobodies with CDR3 lengths of eight aa (upper left), 11 aa (upper middle), and 14 aa (upper right) were overlaid. They were all in intact antigen-nanobody complexes with a great resolution around 3 Å. Unchanged or slightly randomized positions were selectively presented with yellow sticks (lower panel). Created by Pymol. **(C)** Randomization strategy in CDR1, CDR2, and CDR3, respectively. The comparison of relative amino acid frequencies was performed between natural (N) and designed (D) diversity. Cysteine and stop codon were avoided as much as possible. The numbering scheme follows the IMGT nomenclature.

The relationship between hallmarks in FRW2 and CDR3 lengths was taken into consideration. It revealed that F42 and F52 appeared mainly in CDR3 lengths > 14 aa, while Y42 and L50 occurred in CDR3 lengths ≤ 14 aa (Figure 3A), which was the composition of our scaffold. Thus, we chose to take three different lengths (8, 11, or 14 aa) to cover structural diversity for antigen binding selectivity. A constant length of eight amino acids was decided for CDR1 and CDR2. We analyzed the deposited nanobodies via structure superposition. To protect the hydrophobic core and thereby maintain scaffold stability, some aromatic or aliphatic CDR residues (I29, I56, T65, and Y117) contributing to the formation of the hydrophobic core were retained or slightly randomized (Figure 3B). To create the flexibility of CDR loops, several glycine or alanine residues (G27, A35, G63, and A105) were also kept or slightly randomized. It is reported that, irrespective of the region length, CDR2 has the highest diversity among all other regions, followed by CDR1 in VHHs (Li et al., 2016), so randomizations were introduced across the three CDRs. To raise the probability of producing diverse and high-affinity antibodies, a naturally occurring nanobody repertoire was used to mimic and rationally control natural diversity at each position. Eventually, we finished establishing the randomization strategy based on the Hu6B9 scaffold (Figure 3C).

### 3.2 Library construction

**FIGURE 4:**
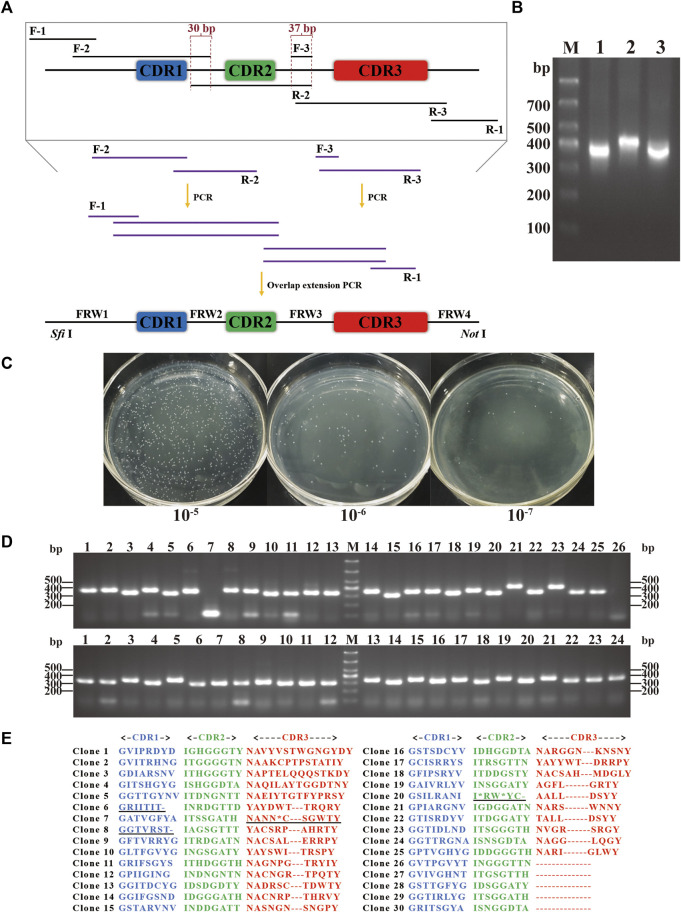
Construction of the synthetic library. **(A)** Schematic representation of the synthetic DNA library. Sequence diversity was introduced using primer pairs of F-2/R-2 and F-3/R-3, while structure diversity was included in R-3 primers. *Sfi* Ⅰ and *Not* Ⅰ enzymatic digestion sites were added using F-1/R-1 primer pair. Overlap extension PCR was performed to assemble the full-length DNA library with digestion sites. **(B)** Nucleic acid electrophoresis of the full-length DNA with digestion sites. Lanes 1, 2, and 3 showed DNA with different CDR3 lengths of 8, 11, and 14 aa. **(C)** The library size was measured by serial dilution. **(D)** The insertion rate was measured by PCR and electrophoresis. 50 clones were randomly picked, and 48 were positive. **(E)** The correctness rate was calculated by sequencing. 30 positive clones were randomly chosen, and 21 clones were correct and non-redundant in CDRs.

Through the *in silico* analysis previously described, primers involving the desired structure and sequence diversity were synthesized. Based on the scaffold, the primers were assembled by performing a few cycles of PCR to build a DNA library encoding the full-length nanobodies (Figures 4A, B). The DNA library was digested and ligated into pCANTAB 5E phagemid vector, which was subsequently electroporated more than 50 times into *E. coli* TG1 cells, resulting in the generation of the synthetic library (Figure 4C). The positive rate of the library was first evaluated. Two out of 50 randomly picked clones were negative, indicating an insertion rate of 96% (Figure 4D). We then evaluated the correctness rate by sequencing 30 inserts. It showed that there were nine incorrect inserts (stop codon, base missing, base shifting, or large region missing), and none of the identical sequences were found (Figure 4E), indicating a correctness rate of 70%. Meanwhile, the amino acid distribution at every CDR was in good consistency with the design. All together, we successfully constructed a synthetic library of humanized nanobodies with an effective size of 3.7 × 10^9^.

### 3.3 Library validation

eGFP, a mutant of GFP, is the most widely used in life science research as an intracellular reporter. It features enhanced brightness, improved monomeric stability, and an emission peak at around 490 nm (Heim and Tsien, 1996). eGFP-specific nanobodies have been proven to be used as tracers for intracellular imaging and numerous other applications (Chen et al., 2023). Moreover, it is demonstrated that the nanobodies enable structural rearrangements in the chromophore environment and thermodynamical stabilization of conformational states of GFP, thus modulating GFP fluorescence (Kirchhofer et al., 2010). Here, prior to identifying the desirable IL-6 binding nanobodies, the library was screened against eGFP. We sought to evaluate whether our library would produce specific and functional eGFP binders that could modulate the fluorescence and be readily detected by instruments.

**FIGURE 5:**
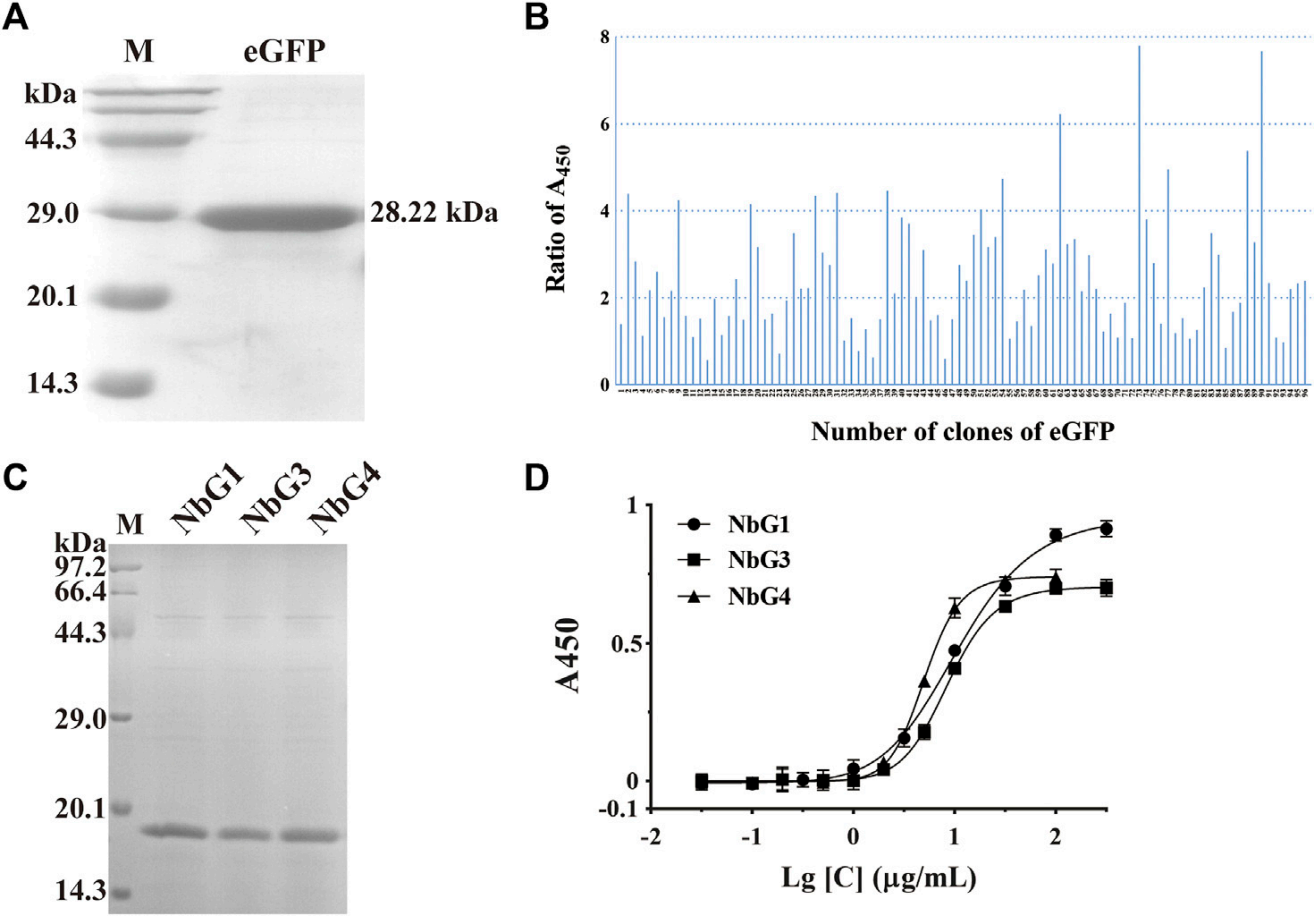
Identification of nanobodies targeting eGFP. **(A)** SDS-PAGE of the purified recombinant antigen protein of eGFP, stained with Coomassie Blue. **(B)** Identification of positive clones by phage ELISA. **(C)** SDS-PAGE of the purified recombinant nanobodies, stained with Coomassie Blue. **(D)** Binding affinity was detected by indirect ELISA.

Recombinant eGFP, with a C-terminus His tag and a molecular weight of 28.22 kDa, was expressed in *E. coli* BL21 (DE3) and purified over the Nickle column. SDS-PAGE analysis of the recombinant eGFP confirmed a purity of over 95% and a high expression of beyond 10 mg/L (Figure 5A). After seven rounds of panning, 51 positive clones out of 96 were shown to detect eGFP by phage ELISA (Figure 5B). We then randomly picked some positive clones, and three non-redundant nanobodies were obtained. All three nanobodies were expressed and soluble in the supernatant. After purification, the recombinant nanobodies were separated by SDS-PAGE and stained with Coomassie Blue. It showed that the purity of these nanobodies was more than 90% (named NbG1, NbG3, and NbG4) (Figure 5C). Subsequently, indirect ELISA was performed, and their binding affinity was identified. They possessed comparable binding strengths to eGFP, ranging from 100 to 1,000 nM (Figure 5D).

**FIGURE 6:**
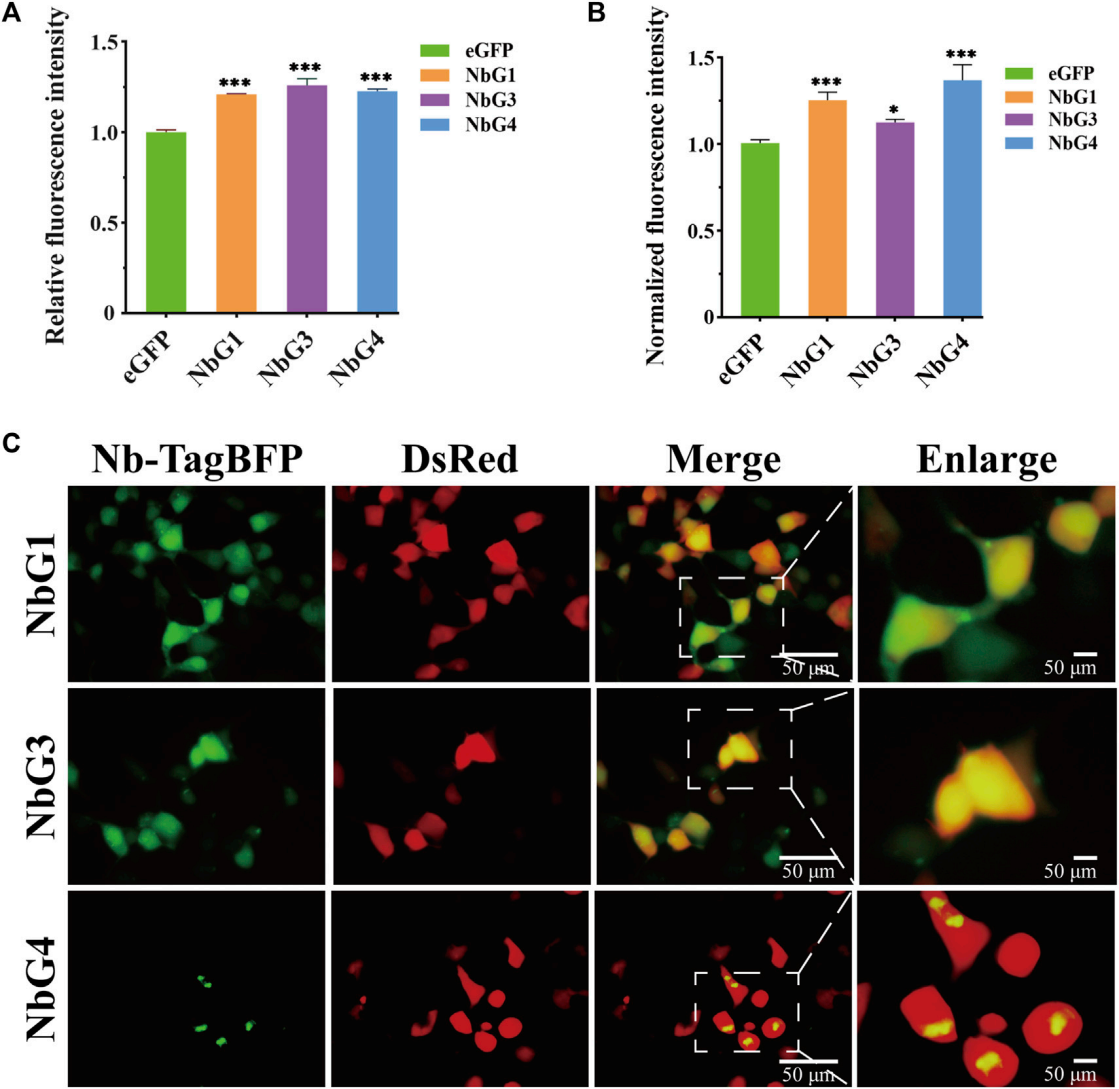
Characterization of nanobodies targeting eGFP. **(A)** Relative fluorescence intensity of eGFP *in vitro*. 6 μM corresponding GFP binding proteins (NbG1, NbG3, or NbG4) were mixed with purified eGFP at an equal concentration in a microplate. The fluorescence intensity of eGFP was quantified using a microplate reader. **(B)** Normalized fluorescence intensity of eGFP intracellularly. Nb-TagBFP, DsRed, and pEGFP plasmids were transiently co-transfected into HEK293T cells. The signal intensities of eGFP and DsRed were acquired using flow cytometry, and eGFP signal normalized to DsRed signal. **(C)** Representative images of Nb-TagBFP expression in HEK293T cells. Nb-TagBFP and DsRed plasmids were transiently co-transfected into HEK293T cells. Both NbG1 and NbG3 exhibited diffuse fluorescent signal, expected of stable and soluble proteins intracellularly. NbG4 exhibited strongly aggregated fluorescent signal, suggestive of intracellular instability.

To measure GFP fluorescence properties that were altered by nanobodies, we mixed eGFP and nanobodies in a plate, and the fluorescence emission at 490 nm was read. Strikingly, it was identified that all nanobodies had a significant effect on enhancing the fluorescence emission of eGFP (Figure 6A). To investigate whether selected nanobodies could still function in a complex intracellular environment, the fluorescence modulation was measured by flow cytometry after the coexpression of the eGFP nanobody and eGFP in HEK293T cells. A comparable effect on fluorescence intensity was also observed (Figure 6B). Since these enhancers still exerted function intracellularly, we were curious whether they were intracellularly stable in HEK293T cells. We cloned nanobodies as fluorescent protein fusions and coexpressed them with DsRed fluorescent protein in 293T cells. Fluorescent imaging revealed that both NbG1 and NbG3 exhibited diffuse fluorescent signal, expected of stable and soluble expression intracellularly (Figure 6C), indicating the potential to detect intracellular proteins (Beghein and Gettemans, 2017). However, NbG4 exhibited fluorescent signal of strong aggregation, suggestive of intracellular instability and inaccessibility to the application of intrabody (Messer and Butler, 2020). All together, NbG1 and NbG3, as stable binders, can increase the fluorescent intensity of eGFP *in vitro* and intracellularly.

### 3.4 Identification and verification of IL-6-specific humanized nanobodies

**FIGURE 7:**
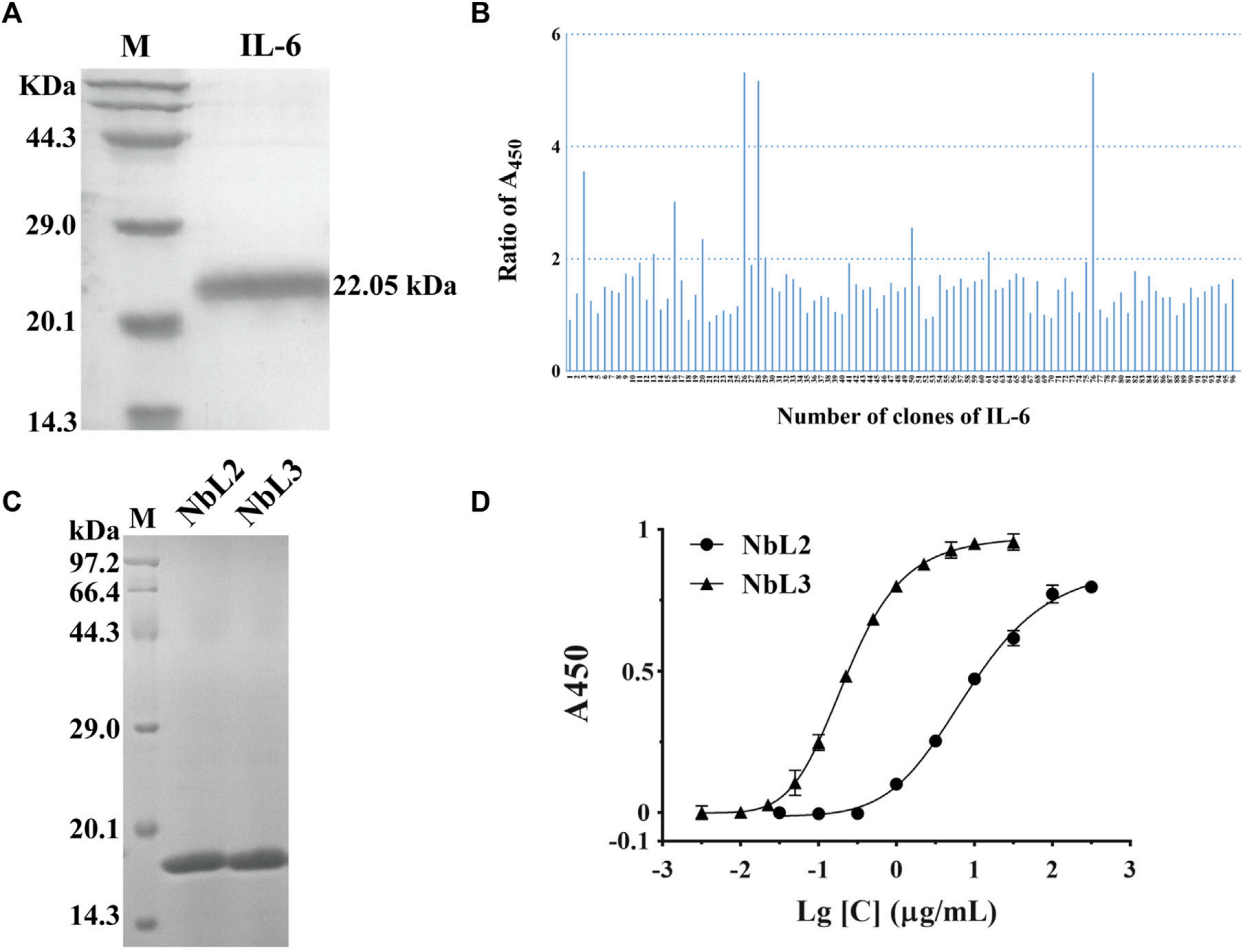
Identification of humanized nanobodies targeting IL-6. **(A)** SDS-PAGE of the purified recombinant antigen protein of IL-6, stained with Coomassie Blue. **(B)** Identification of positive clones by phage ELISA. **(C)** SDS-PAGE of the purified recombinant nanobodies, stained with Coomassie Blue. **(D)** Binding affinity was detected by indirect ELISA.

In analogy to the successful selections against eGFP, we carried out the second screening against IL-6. We first prepared recombinant IL-6 with a N-terminus His tag and a molecular weight of 22.05 kDa. SDS-PAGE analysis confirmed that the purity of recombinant IL-6 was over 95% (Figure 7A). Five rounds of selection were used to identify clones that bound to IL-6.10 out of 96 clones were identified as positive by phage ELISA (Figure 7B). We then randomly picked some positive clones, and two non-redundant nanobodies were obtained. Both were expressed and soluble in the supernatant, followed by purification over the Nickle column. The recombinant nanobodies were analyzed by SDS-PAGE, indicating their purity was more than 95% (named NbL2 and NbL3) (Figure 7C). Subsequently, their binding affinity was detected by performing an indirect ELISA. Notably, NbL2 possessed a high affinity of 22.16 nM (Figure 7D).

**FIGURE 8:**
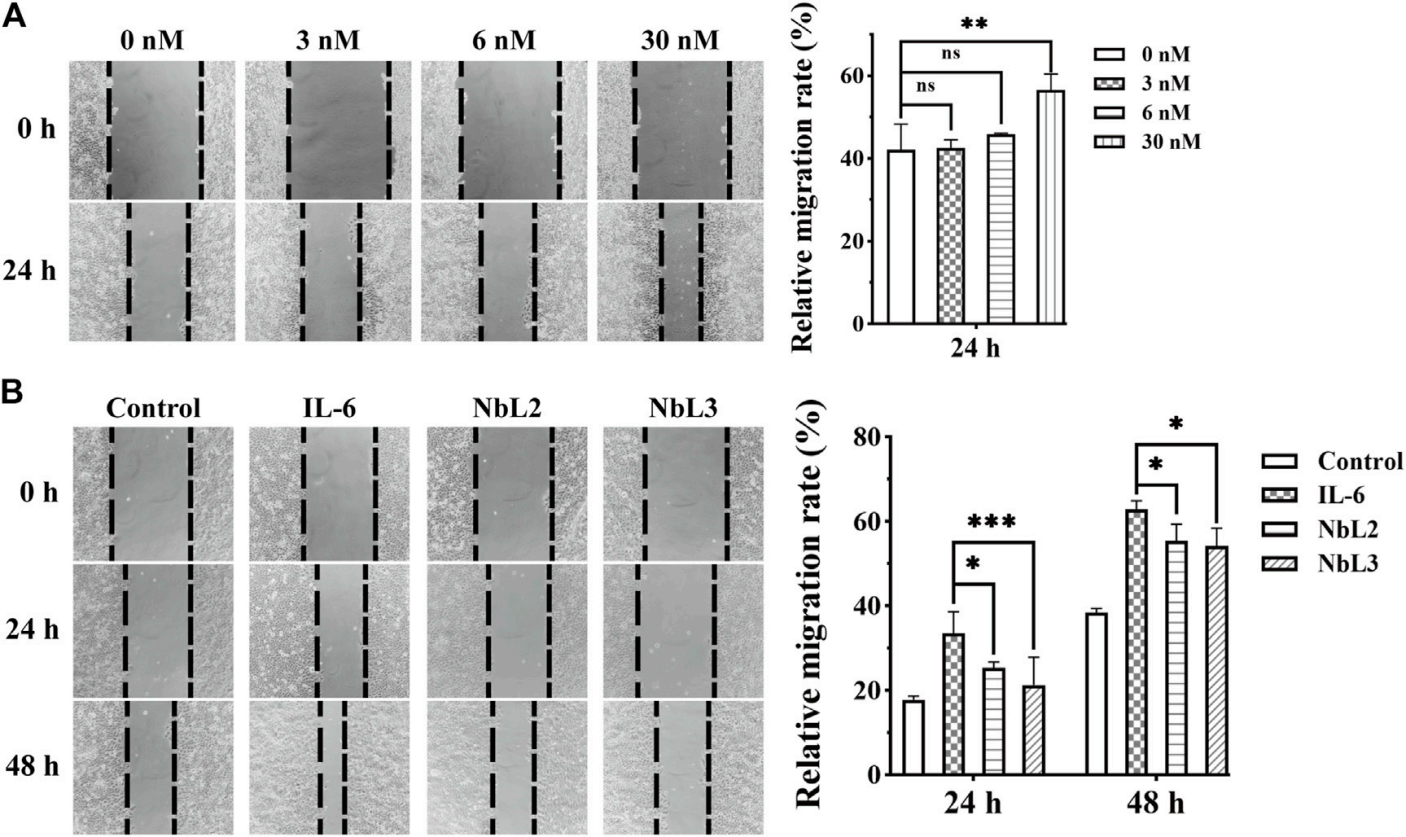
Migration analysis of MCF-7 cells. **(A)** The effect of IL-6 on the migration of MCF-7 cells. The wound healing assay was carried out at an increasing concentration of IL-6 (left panel). The scratch area was marked and captured on 0 and 24 h. The migration rate of MCF-7 cells was quantified by the measurements of the repaired scratch area (right panel). Data were expressed as means ± SEM. **p < 0.01, IL-6 treatment vs*.* no IL-6 group. ns indicates no significance. **(B)** The effect of anti-IL-6 nanobodies on IL-6-enhanced migration of MCF-7 cells. The wound healing assay was performed at 30 nM IL-6 in combination with 1 μM NbL2 or 0.1 μM NbL3 (left panel). Control group was not subjected to any treatments. IL-6 group was only treated with 30 nM IL-6. NbL2 and NbL3 groups were treated with IL-6 in combination with corresponding nanobodies. The scratch area was marked and captured on 0, 24, and 48 h. The migration rate of MCF-7 cells was quantified by the measurements of the repaired scratch area (right panel). Data were expressed as means ± SEM. *p < 0.05, ***p < 0.001, NbL2 or NbL3 group vs*.* IL-6 group.

Breast cancer is the most common type of cancer and the third leading cause of cancer-associated mortality among females in the world (Siegel et al., 2023). The carcinoma cells of the luminal A molecular subtype are characterized by positive hormonal receptors (HRP+) and negative human epidermal growth factor receptors (HER2‒). HRP + breast cancer with positive metastatic lymph nodes shows a high risk of tumor relapse (Nouh et al., 2004). IL-6 activates the signal transduction of STAT3 through a hexameric complex of IL-6, IL-6R, and glycoprotein 130 (gp130) in MCF-7 cells (Dittmer et al., 2020). MCF-7 belongs to the human HRP + -breast cancer cell line, and IL-6 significantly enhances its invasiveness with a low concentration (Ibrahim et al., 2016), leading to an increased risk of death in patients. Thus, we sought to assess the blocking activity of NbL2 and NbL3 by wound healing assay. The effective concentration of the recombinant IL-6 on MCF-7 was explored in the first place, and 30 nM IL-6 was determined as the stimulus to enhance the migration of MCF-7 (Figure 8A). Thereafter, 1.0 μM NbL2 or 0.1 μM NbL3, together with 30 nM recombinant IL-6, were chosen to influence the IL-6-enhanced migration on MCF-7. It revealed that both significantly inhibited the IL-6-augmented migration in 24 and 48 h (Figure 8B), indicating a potent activity of blocking the binding of IL-6 to IL-6R on MCF-7.

**FIGURE 9:**
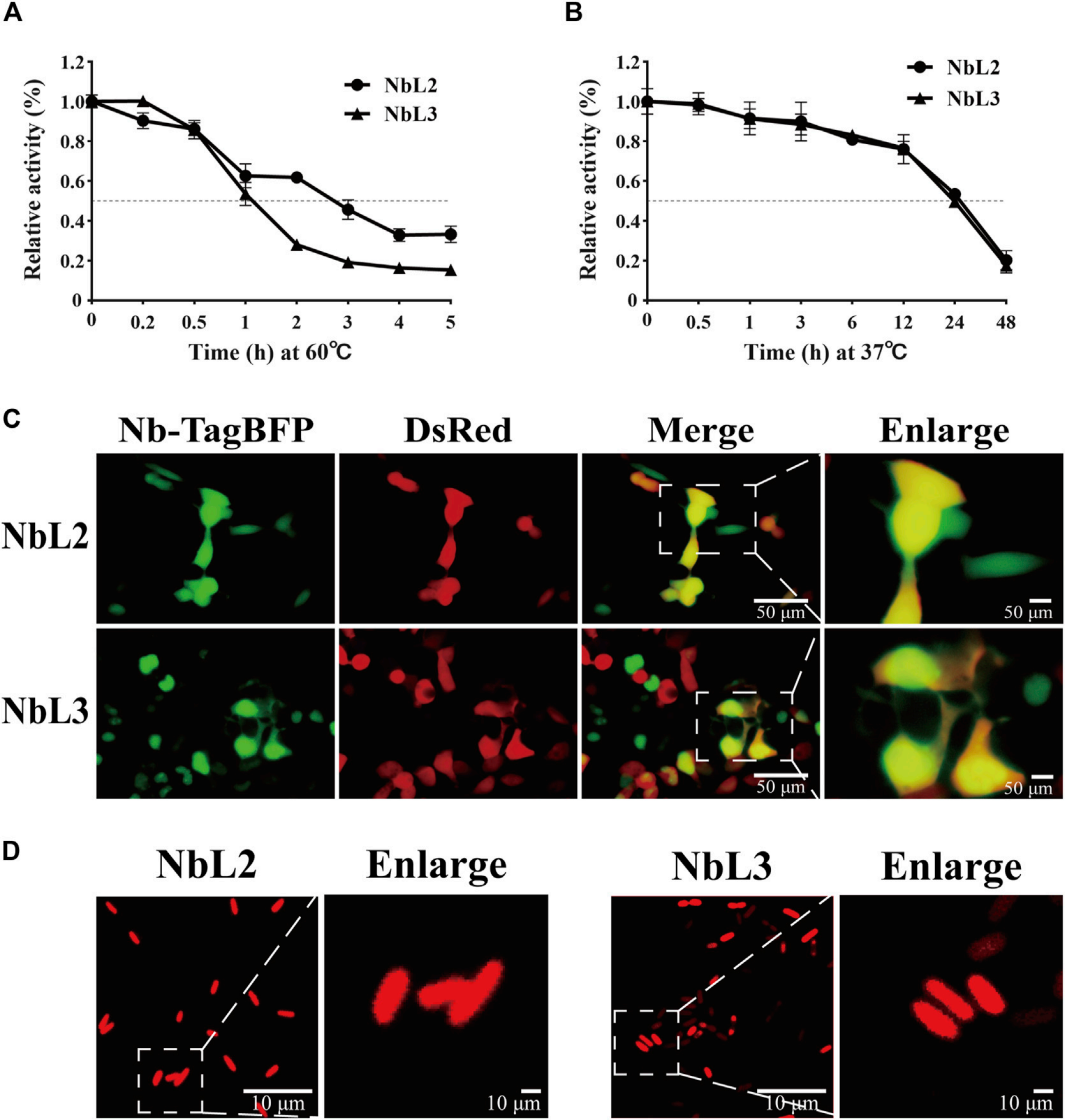
Characterization of humanized nanobodies targeting IL-6. **(A)** Thermostability analysis of NbL2 and NbL3 at 60°C. Binding activity was determined after treatment at 60°C. **(B)** Thermostability analysis of NbL2 and NbL3 at 37°C. Binding activity was determined after treatment at 37°C. **(C)** Representative images of Nb-TagBFP expression in HEK293T cells. Strong and diffuse fluorescent signal was observed. **(D)** Representative images of Nb-mCherry expression in *E. coli*. Nb-mCherry plasmid was transformed into *E. coli* BL21 (DE3). Each image was taken following the protein induction of bacteria cultured from a single colony. Strong and diffuse fluorescent signal was observed.

To verify the application potential of the anti-IL-6 humanized nanobodies, thermostability was assessed by performing indirect ELISA. NbL3 still retained binding activity over 50% after treatment for 1 h at 60°C, in comparison to treatment for nearly 3 h on NbL2 (Figure 9A). The binding activity curves at 37°C were superimposable, indicating a retention of over 50% binding activity after treatment for 24 h (Figure 9B). We then illustrated the intracellular expression patterns by live fluorescence imaging. Both NbL2 and NbL3 exhibited strong and diffuse fluorescent signal, indicative of well-expressed and stable proteins in HEK293T (Figure 9C). This offers a therapeutic modality to deliver nanobodies merged with Fc (Guglielmi et al., 2011) for linking T-cells or NK cells (Bannas et al., 2017). Additional tests of expression in bacteria were further performed. Nanobodies were fused N-terminally to mCherry, and their expression patterns were assessed by confocal microscopy. Both NbL2 and NbL3 exhibited intense and diffuse fluorescent signal (Figure 9D). In this context, their stable and soluble expression may contribute to improved production yields for bacteria-produced nanobody reagents.

## 4 Discussion

Here, we report the construction of the synthetic library of humanized nanobodies based on a novel scaffold, Hu6B9, and the identification of the blocking activity of humanized nanobodies targeting IL-6. The Nb6B9 nanobody was first preferred from a set of nanobody-antigen complexes. After the introduction of five modifications for humanization and stability optimizations, we designed the Hu6B9 scaffold, which is more stable than Nb6B9 and closer to human VH3. To increase the sequence and structure diversity of the library, sequence alignment and structure superposition analysis were performed to raise the probability of producing diverse and high-affinity antibodies, together with ensuring the folding of the hydrophobic core. We then assembled a DNA library by performing a few cycles of PCR using degenerate primers involving the desired rational design. After electroporation more than 50 times, the synthetic library with an effective size of 3.7 × 10^9^ was obtained. To assess its feasibility, the library was screened against eGFP. Selections were carried out, and three eGFP binding nanobodies were obtained. It was demonstrated that two of them were intracellularly stable and functional to enhance the fluorescence intensity of eGFP, indicating that the library is feasible to generate functional nanobodies with good intracellular stability.

After the construction and validation experiments, humanized nanobodies targeting IL-6 were expected to be generated from the library. Selections were performed, and two nanobodies were identified. Notably, NbL3 possessed a high affinity of 22.16 nM. We then explored their blocking activity on MCF-7. It revealed that both significantly inhibited the IL-6-enhanced migration of MCF-7 with NbL3 at a low concentration of 0.1 μM. Thus, our anti-IL-6 humanized nanobodies are identified as potent candidates to block the binding of IL-6 to IL-6R, which provides alternatives for the treatment of IL-6-related diseases. Additionally, they have comparable thermostability to other synthetic nanobodies (Yan et al., 2014). Thermal stability contributes to various processes and applications *in vitro*, such as preservation, shipping, detection, and diagnosis. However, the broadly detailed nanobody stability in an extracellular context does not resemble the cytoplasmic environment. Nanobodies are readily amenable in various expression systems owing to their strong resistance to the reducing environment of the cytoplasm (Wesolowski et al., 2009) and improved proteolytic stability (Harmsen et al., 2006; Hussack et al., 2011), leading to successful applications in both oral and bacterial delivery systems for tumor treatment (Chowdhury et al., 2019; Wang et al., 2024). In contrast, antibodies with intracellular aggregation may put cellular survival under stress, which is investigated by the aggregation of a range of aggregation-prone factors, including prion protein (Abskharon et al., 2014) and alpha synuclein (Chatterjee et al., 2018). Therefore, following the blocking activity assay on MCF-7 and thermostability characterization, we explored the intracellular stability of the anti-IL-6 nanobodies. Their intense and diffuse fluorescence signal demonstrated that they were highly stable and soluble in both bacteria and HEK293T, expanding the range of potential applications, such as fusion expression with Fc in the eukaryotic system and delivery via the oral cavity or safe microorganisms. All together, we obtained potent IL-6 binders with significant blocking activity and good stability, providing potential candidates for IL-6-related disease diagnosis and treatment. The synthetic library provides a novel platform for screening functional and humanized nanobodies against various antigens.

GFP binding nanobodies could compete for overlapping epitopes, leading to distinct effects on fluorescence enhancement or minimization (Kirchhofer et al., 2010). After the determination of crystal structures and the elucidation of molecular mechanisms, it was revealed that both the enhancer and the minimizer recognized slightly overlapping epitopes and induced local structure rearrangements across the GFP chromophore environment. Thus, for the initial goal, the library was expected to produce a pair of eGFP binding nanobodies that could augment or diminish the fluorescence. Unfortunately, it produced nanobodies that only augmented the fluorescence of eGFP. It is observed that the entire FRW2 area of the GFP enhancer participates in GFP recognition, where Y37 directs the major interaction (Kirchhofer et al., 2010). Notably, the short CDR3 loop of the enhancer is stretched out, making the FRW2 accessible to solvent. In contrast, the minimizer normally folds its CDR3 over the FRW2 and binds GFP with its long CDR3 loop. All together, a possible reason for the absence of minimizers here is attributed to the absence of elongated CDR3 in our library. Nevertheless, the selected GFP fluorescence enhancers in our work would improve the resolution of *in vivo* structured illumination (Kner et al., 2009) and *in vitro* labeling approaches (Platonova et al., 2015), and benefit any applications that require fluorescence enhancement by the coexpression of the enhancers. Additionally, these nanobodies may provide theoretical support for the brightness improvement of eGFP.

In this work, the Nb6B9 nanobody was first used as the template to build a scaffold. We introduced humanization optimization, together with stability improvement, in the template, and the Hu6B9 scaffold was obtained with greater humanization and stability, demonstrating a successful strategy to create the nanobody scaffold. There have been extensive investigations into thermal stabilization, including improving hydrophobic packing and optimizing surface charge (Kunz et al., 2017). It is noteworthy that there is a distinction between thermostability and structural conservation. As summarized previously, mutations Q49G and R50L increase thermostability and unexpectedly, our result confirmed that again. However, we found slight aggregation of the nanobodies at a high concentration and even at a low temperature (4°C). The partial consensus framework indicates highly conserved positional residues of nanobody structures, where R50 is included. This may explain why the nanobodies showed slight aggregation but good stability. To administer nanobodies in diagnostics and therapeutics, their ability to be preserved at high concentrations is necessary. Thus, the mutation R50L should not be favored.

To establish the randomization strategy, we analyzed the relationship between the hallmarks in FRW2 and the lengths of CDR3. To our knowledge, this is for the first time used to guide the construction of a synthetic nanobody library, providing theoretical support for the randomization of CDR3 lengths. Synthetic degenerate primers are a cost-effective means to assemble a library, and we succeeded in identifying high-affinity and functional nanobodies using this library. However, degenerate primers cannot take full control of the desired codon frequency, and are susceptible to cysteine and stop codon, leading to a large proportion of incorrectness. Based on the successful screenings of the library, the trinucleotide DNA assembly strategy is preferred to match the desired codon frequency and decrease incorrectness, producing a better synthetic library. Hence, better candidates would be provided to apply for IL-6-associated diagnostics or therapeutics.

## 5 Conclusion

In summary, based on a scaffold optimized for humanization and stability, we developed a synthetic library of humanized nanobodies. It was validated by screening against eGFP to produce highly functional and intracellularly stable nanobodies. Using the library, we identified humanized nanobodies targeting IL-6, which significantly blocked the binding between IL-6 and IL-6R on MCF-7 cells. Additionally, they were thermostable *in vitro* and intracellularly stable in HEK293T and bacteria. These IL-6-binding humanized nanobodies are promising alternatives for IL-6-related disease therapeutics and diagnostics. Moreover, the synthetic library provides a versatile platform for the development of functional and humanized nanobodies against multiple targets.

## Data Availability

The original contributions presented in the study are included in the article/Supplementary Material, further inquiries can be directed to the corresponding authors.
