# Supplementary material for: Development of a synthetic library of humanized nanobodies for targeted IL-6 inhibition
Source: Front Bioeng Biotechnol. 2024 Jul 23;12:1440150. doi: 10.3389/fbioe.2024.1440150 (PMC11300276; doi:10.3389/fbioe.2024.1440150)
Supplement: Supplementary file 1 [file Table1.DOCX]

Supplementary Material

# Supplementary Data

**Supplementary Table 1: The PCR primers used in the construction of the synthetic DNA library.**

| Primer Name | Oligonucleotides Sequence (5**’**→3**’)** | Underline |
| --- | --- | --- |
| F-1 | TTGTTCCAATGAATAAGGCCCAGCCGGCCCAGGTTCAGCTGGT  TGAAAGCGGTGGTGGT | *Sfi* Ⅰ |
| R-1 | GGATACGGCACAAGGAAAAAAGCGGCCGCTGAAGACACGGT  AACCTGTGTACCCTGACC | *Not* Ⅰ |
| F-2 | CAGGTTCAGCTGGTTGAAAGCGGTGGTGGTCTGGTTCAGGCA  GGTGGTTCACTGCGTCTGTCATGTGCAGCAAGCGGTNNYAYY  NYYVRYNNYWAYRNYATGGGTTGGTATCGTCAGGCACCGGGT  AAAGGTCTGGAACTGGTTG | CDR1 |
| R-2 | ATCTGCAGATAAACTGTATTTTTTGCATTATCACGGGAAATAGT  AAAACGACCTTTAACTGAATCGGCATARKNAGTRBYACCRYY  RYBRBYAATGGCCGCAACCAGTTCCAGACCTTTACCCGGTGC  CT | CDR2 |
| F-3 | CCCGTGATAATGCAAAAAATACAGTTTATCTGCAGAT |  |
| R8-3 | TGAAGACACGGTAACCTGTGTACCCTGACCCCARKAMNNMN  BMNNMNNMNNMNBRKYGCAATAATAAACTGCGGTATCTTCC  GGTTTCAGGCTATTCATCTGCAGATAAACTGTATTTTTTGCATT  A | 8 aa CDR3 |
| R11-3 | TGAAGACACGGTAACCTGTGTACCCTGACCCCAATARNBMYN  RBBMNNRNNYSNRYNRYNTGCRTWGCAATAATAAACTGCGG  TATCTTCCGGTTTCAGGCTATTCATCTGCAGATAAACTGTATTTT  TTGCATTA | 11 aa CDR3 |
| R14-3 | TGAAGACACGGTAACCTGTGTACCCTGACCCCAATARNYMNN  MNNMNNMNNMNNMNNMNNMNNMNNMNNTGCATTGCAAT  AATAAACTGCGGTATCTTCCGGTTTCAGGCTATTCATCTGCAGA  TAAACTGTATTTTTTGCATTA | 14 aa CDR3 |
